# Gemcitabine Induces Microvesicle Particle Release in a Platelet-Activating Factor-Receptor-Dependent Manner via Modulation of the MAPK Pathway in Pancreatic Cancer Cells

**DOI:** 10.3390/ijms20010032

**Published:** 2018-12-21

**Authors:** Anita Thyagarajan, Sayali M. Kadam, Langni Liu, Lisa E. Kelly, Christine M. Rapp, Yanfang Chen, Ravi P. Sahu

**Affiliations:** Department of Pharmacology and Toxicology, Boonshoft School of Medicine at Wright State University, Dayton, OH 45435, USA; anita.thyagarajan@wright.edu (A.T.); kadam.8@wright.edu (S.M.K.); liu.106@wright.edu (L.L.); lisa.kelly@wright.edu (L.E.K.); christine.rapp@wright.edu (C.M.R.); yanfang.chen@wright.edu (Y.C.)

**Keywords:** pancreatic cancer, platelet-activating factor-receptor, gemcitabine, microvesicle particles

## Abstract

Studies, including ours, have shown that pro-oxidative stressors, such as chemotherapeutic agents, generate oxidized lipids with agonistic platelet-activating factor (PAF) activity. Importantly, recent reports have implicated that these PAF-agonists are transported extracellularly via microvesicle particles (MVPs). While the role of PAF-receptor (PAF-R) has been implicated in mediating chemotherapy effects, its significance in chemotherapy-mediated MVP release in pancreatic cancer has not been studied. The current studies determined the functional significance of PAF-R in gemcitabine chemotherapy-mediated MVP release in human pancreatic cancer cells. Using PAF-R-expressing (PANC-1) and PAF-R-deficient (Hs766T) cells, we demonstrate that gemcitabine induces MVP release in a PAF-R-dependent manner. Blocking of PAF-R via PAF-R antagonist or inhibition of MVP generation via inhibitor of acid sphingomyelinase (aSMase) enzyme, significantly attenuated gemcitabine-mediated MVP release from PANC-1 cells, however, exerted no effects in Hs766T cells. Notably, MVPs from gemcitabine-treated PANC-1 cells, contained a measurable amount of PAF-agonists. Mechanistically, pretreatment with ERK1/2 or p38 inhibitors significantly abrogated gemcitabine-mediated MVP release, indicating the involvement of mitogen-activated protein kinase (MAPK) pathway in PAF-R-dependent gemcitabine-mediated MVP release. These findings demonstrate the significance of PAF-R in gemcitabine-mediated MVP release, as well as the rationale of evaluating PAF-R targeting agents with gemcitabine against pancreatic cancer.

## 1. Introduction

Exposure to pro-oxidative stressors, including therapeutic agents, has been shown to generate oxidized lipids with platelet-activating factor (PAF) agonists’ activity from various cell types, including tumor cells [1,2,3,4,5,6,7]. In particular, tumor cells expressing PAF-receptor (PAF-R) exhibit enhanced levels of PAF-agonists generation in response to chemotherapy [5,6]. While multiple signaling mechanisms have been proposed in mediating PAF-R-dependent effects of therapeutic agents in malignant cells [5,6,7,8,9,10,11], it is not clear if PAF-R-dependent effects in response to stimuli, such as during chemotherapy, are mediated directly or indirectly.

Growing evidence supports the crucial roles of microvesicle particles (MVPs), an extracellular vesicles in mediating the biological activities of cells in response to stimuli, including cancer therapy [12,13,14,15]. MVPs are small membrane-bound nanosized (10–100 nm) particles which are released by various cell types, including tumor cells, and contain a variety of bioactive substances, including lipids [14,15,16,17,18]. Thus, ongoing efforts are directed toward defining the functional role, as well as mechanisms of MVPs in disease pathophysiologies, including cancers, to devise better therapeutic strategies against these ailments. 

The recent reports from our group have demonstrated that PAF-agonists generated in response to the environmental stressor, ultraviolet B (UVB), are extracellularly transported via MVP [16,17]. Notably, UVB exposure resulted in MVP release in a dose-dependent manner from PAF-R-expressing human keratinocyte HaCaT cells, and human skin explants [16,17]. This UVB-induced effect on MVP release is mimicked by a known PAF-R agonist, carbamoyl-PAF (CPAF) [17]. Importantly, exposure of UVB or CPAF to PAF-R-expressing (KBP) and PAF-R-deficient (KBM) human epidermoid cells induced significant levels of MVP release selectively from KBP cells, compared to KBM cells, indicating the necessity of PAF-R in mediating UVB/CPAF-induced MVP release [17]. 

Studies, including ours, have demonstrated that PAF-R activation plays critical roles in various disease pathophysiologies, including cancer growth [1,2,3,4,5,6,7,8,19]. While effects of PAF-R have been shown in various cancer models [5,6,7,8,9,10,11], little is known about its effect in pancreatic cancer models. Pancreatic cancer, like other major human cancers, has poor prognosis, and it is difficult to treat this malignancy, with high mortality rates in the United States [20,21,22,23]. Notably, the standard gemcitabine chemotherapy alone has been shown to exert a low response rate, and when combined with other agents, exhibited mixed to slightly improved responses [24,25,26,27,28]. These findings indicate the critical need to identify novel approaches/strategies for the treatment of pancreatic cancer. 

The current studies sought to determine the role and mechanism of PAF-R in gemcitabine-mediated MVP release in human pancreatic cancer cells. Our studies using PAF-R-expressing (PANC-1) and -deficient (Hs766T) cells demonstrate that gemcitabine induces MVP release in a PAF-R dependent manner, in a process blocked by PAF-R antagonist or acid sphingomyelinase (aSMase) inhibitor. Mechanistically, gemcitabine-induced MVP release was significantly attenuated by ERK1/2 and p38 inhibitors, indicating the role of MAPK pathway in PAF-R-dependent gemcitabine-induced MVP release. 

## 2. Results

### 2.1. Gemcitabine Treatments Release MVP in a PAF-R-Dependent Manner

**Figure 1:**
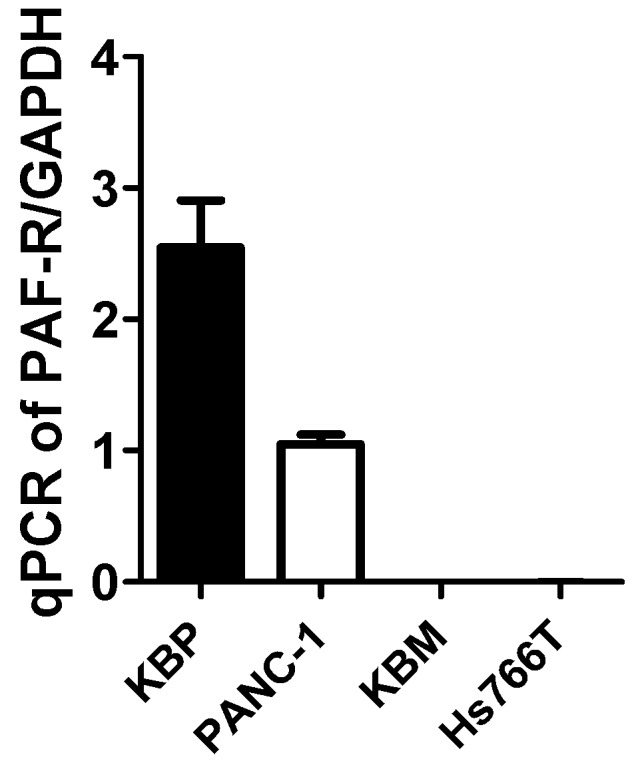
Evaluation of platelet-activating factor-receptor (PAF-R) mRNA expression. qPCR analysis demonstrated that PANC-1 human pancreatic cancer cells express, and Hs766T cells lack, PAF-R expression. PAF-R-expressing human epidermoid KBP, and deficient KBM cells, were used as positive and negative controls.

**Figure 2:**
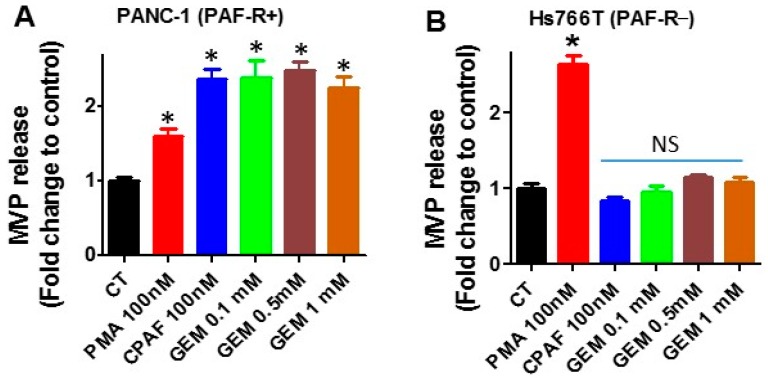
Effect of gemcitabine on microvesicle particle (MVP) secretes. (**A**) PANC-1 and (**B**) Hs766T cells were treated with or without phorbol 12-myristate 13-acetate (PMA), carbamoyl-PAF (CPAF), or gemcitabine (GEM) at given doses. After 4 h of incubation, MVPs were isolated and analyzed. Data are representative of mean ± SD of three independent experiments, normalized per 1 × 10^6^ cells. The sign (* = *p* < 0.05) denotes statistically significant differences from control (CT), and NS denotes a non-significant difference from CT.

Our first studies using PAF-R-expressing PANC-1 and PAF-R-deficient Hs766T cells (Figure 1) determine effects of PAF-R expression on gemcitabine-mediated MVP release. To that end, PANC-1 and Hs766T cells were treated with or without various doses of gemcitabine (GEM; 0.1, 0.5, and 1 mM), and incubated for 4 h as per our previous reports [17]. Cells treated with 0.1% ethanol served as a negative control, and with CPAF (100 nM, for PAF-R-expressing) served as positive control. However, treatment of phorbol 12-myristate 13-acetate (PMA; 100 nM, also known as TPA), a PAF-R-independent PKC agonist, served as a positive control for PAF-R-deficient cells, which also induces MVP release in PAF-R-expressing cells [29]. We observed that gemcitabine resulted in significant levels of MVP release in PANC-1 cells, similar to that observed with PMA or CPAF treatments compared to normal control (Figure 2A). Notably, only PMA, but not gemcitabine or CPAF treatments, resulted in MVP release in Hs766T cells, compared to control group (Figure 2B). These findings indicate the potential role of the PAF-R in gemcitabine-mediated MVP release. These findings further characterized, and defined, the role of cellular PAF-R in MVP release in response to stimuli similar to, as described [16,17]. Since, we did not observe a dose-dependent effect of gemcitabine on MVP release (Figure 2A), and we chose 0.1 mM dose of gemcitabine for our next experiments.

### 2.2. Blockade of PAF-R Attenuate Gemcitabine-Induced MVP Release

**Figure 3:**
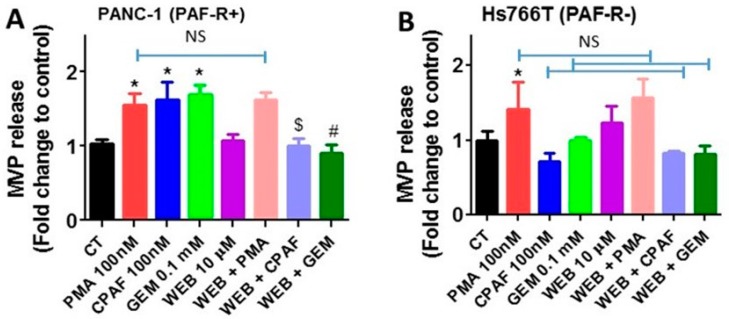
Effect of PAF-R antagonist on gemcitabine-induced MVP release. (**A**) PANC-1 and (**B**) cells were pretreated with PAF-R antagonist, WEB2086 (10 µM, 1 h) followed by treatments with or without PMA, CPAF, or GEM at given doses. After 4 h of incubation, MVPs were isolated and analyzed. Data are representative of mean ± SD of three independent experiments, normalized to 1 × 10^6^ cells. The sign (* = *p* < 0.05) denotes statistically significant differences between control (CT) vs. PMA, CPAF, or GEM groups, and (^$^ = *p* < 0.05) between CPAF vs. WEB + CPAF, and (^#^ = *p* < 0.05) between GEM vs. WEB + GEM groups. The sign NS denotes non-significant differences compared to PMA, CPAF, or GEM groups.

Previous studies, including ours, have shown that PAF-R antagonist attenuates PAF-R-mediated effects of various stimuli, including antitumor agents [7,29,30,31]. Thus, our next studies determined the effect of a PAF-R antagonist, WEB2086, on gemcitabine-induced MVP release. For this, PANC-1 and Hs766T (for control) cells were pretreated with WEB2086 (10 µM) for 1 h, followed by treatments with or without gemcitabine (0.1 mM), PMA (100 nM), or CPAF (100 nM), and incubated for 4 h. We observed that WEB2086 significantly attenuated gemcitabine- and CPAF-mediated, but not PMA-induced, MVP release in PANC-1 cells (Figure 3A). Importantly, WEB2086, which blocked CPAF-mediated MVP release, did not exert any effects on PMA-induced MVP release in Hs766T cells (Figure 3B). These findings further confirmed that PAF-R expression augments gemcitabine-mediated MVP release.

### 2.3. Inhibition of Acid Sphingomyelinase Enzyme Blocks Gemcitabine-Induced MVP Release

**Figure 4:**
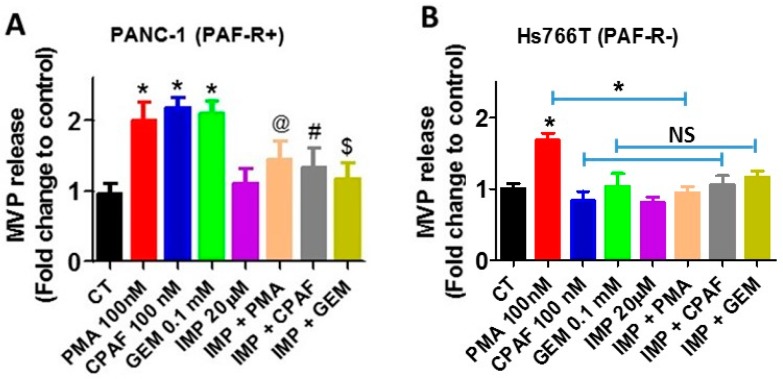
aSMase inhibition abrogates GEM-induced MVP release. (**A**) PANC-1 and (**B**) Hs766T cells were pretreated with aSMase inhibitor, imipramine (20 µM, 1 h), followed by treatments with or without PMA, CPAF, or GEM at given doses. After 4 h of incubation, MVPs were isolated and analyzed. Data are representative of mean ± SD of three independent experiments, normalized per 1 × 10^6^ cells. The signs (* = *p* < 0.05) denote statistically significant differences between control (CT) vs. PMA, CPAF, or GEM groups, and (^@^ = *p* < 0.05) between PMA vs. IMI + PMA, (^#^ = *p* < 0.05) between CPAF vs. IMI + CPAF, and (^$^ = *p* < 0.05) between GEM vs. IMI + GEM group. NS denotes non-significant differences compared to CPAF or GEM groups.

Activation of acid sphingomyelinase enzyme (aSMase) induces MVP generation, and its inhibition via an aSMase-specific inhibitor, imipramine, has been shown to block MVP release [32]. Our next studies determined if gemcitabine-mediated MVP release occurs via the aSMase pathway. To that end, PANC-1 and Hs766T cells were pretreated with imipramine (20 µM) for 1 h, followed by treatments with or without gemcitabine (0.1 mM), PMA (100 nM), or CPAF (100 nM) for 4 h, as described. We observed that imipramine blocked not only gemcitabine, but also PMA and CPAF-mediated MVP release in PANC-1 (Figure 4A) or Hs766T (Figure 4B) cells, indicating the role of aSMase in MVP release. 

### 2.4. MVPs from Gemcitabine-Treated Cells Contain PAF-R Agonists

**Figure 5:**
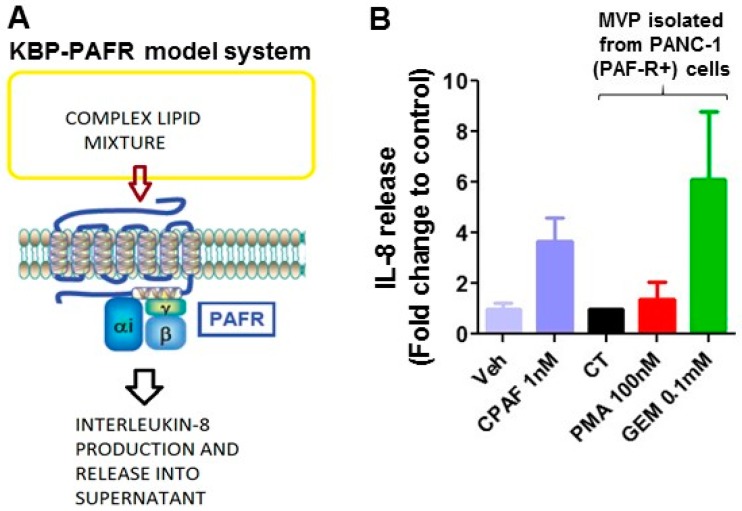
GEM-secreted MVPs contain PAF-R agonists. (**A**) Schematic representation of KBP-PAFR model system. (**B**) In this assay, human KBP (PAF-R+) cells were treated with lipid extracts from MVPs isolated from control (CT) PMA (100 nM) or gemcitabine (0.1 mM)-treated PANC-1 cells. KBP cells treated with vehicle (Veh) or CPAF (1 nM) served as negative and positive controls. After 6 h of incubation, supernatants were collected, and IL-8 levels (mean ± SD, pg/MVP from 1 × 10^10^ cells, from 3 independent experiments) were measured as a surrogate marker for PAF-R agonist generation.

Multiple studies have demonstrated that MVPs contain bioactive components, including lipids [16,17,18]. As therapeutic agents, including chemotherapeutic agents, generate PAF-R agonists from tumor cells [6,7], we next tested if MVPs released by gemcitabine contain PAF-R agonists. To that end, PANC-1 cells were treated with or without gemcitabine (0.1 mM) or PMA (100 nM) as a positive control, and incubated for 4 h. MVPs were isolated from various treatments, and lipids extracted per our previous reports [4,6] were added separately to PAF-R-expressing KBP and -deficient KBM cells. These cells were also treated with or without CPAF (1 nM). After 6 h of incubation, supernatants were analyzed for interleukin 8 (IL-8) as a surrogate marker of PAF-R agonists, as per previous reports [4,6]. This is a well-established methodology to define the PAF-R agonistic activity of various stimuli (Figure 5A). We observed that MVPs released as a result of gemcitabine contain PAF-R agonists comparable to the level of CPAF added directly to KBP cells (Figure 5B), and these did not induce IL-8 release from KBM cells. 

### 2.5. MAPK Pathway Mediates PAF-R-Dependent Gemcitabine-Induced MVP Release

**Figure 6:**
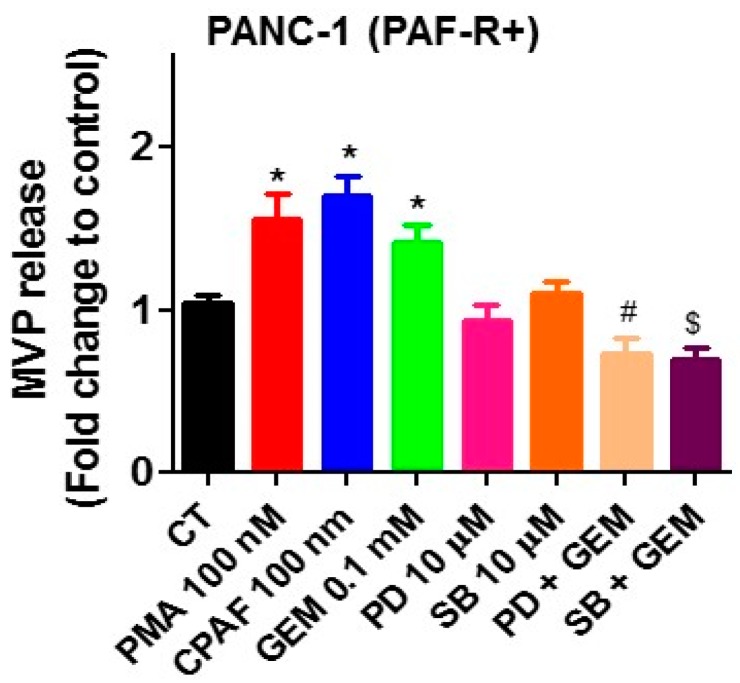
MAPK pathway inhibitors block GEM-induced MVP release. PANC-1 cells were pretreated with inhibitors of ERK1/2 (PD98059;10 µM) or p38 (SB203580; 10 µM) for 1 h, followed by treatments with or without PMA, CPAF or GEM at given doses. After 4 h, MVPs were isolated and analyzed. Data are representative of mean ± SD of three independent experiments, normalized to 1 × 10^6^ cells. The sign (* = *p* < 0.05) denotes statistically significant differences between CT and PMA, CPAF, or GEM groups. (^#^ = *p* < 0.05) between PD vs. PD + GEM, and (^$^ = *p* < 0.05) between SB vs. SB + GEM group.

**Figure 7:**
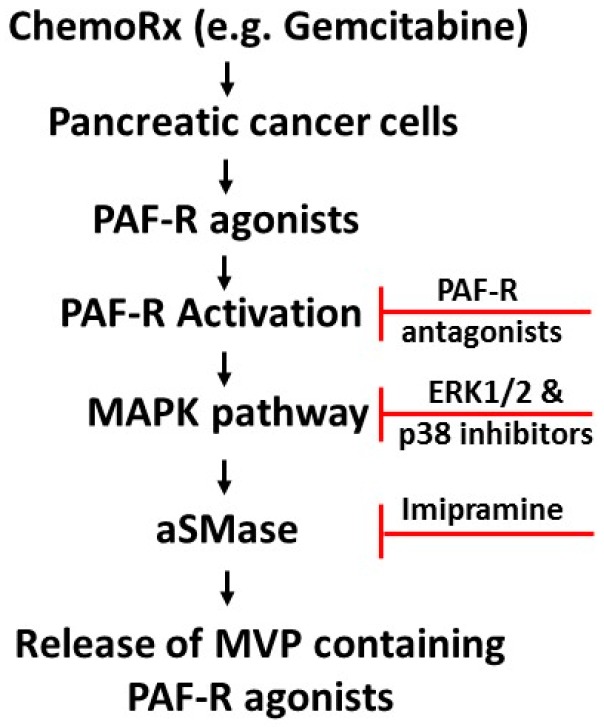
Proposed model of ChemoRx-induced effects on PAF-R-dependent gemcitabine-induced MVP release. The sign 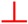 denotes inhibition.

Several cellular signaling pathways have been implicated in mediating PAF-R-dependent effects in response to stimuli such as chemotherapy [8,9,10,11]. As MAPK pathways play a central role in several cellular activities of cancer cells, we evaluated MAPK pathway, in particular, roles of extracellular signal-regulated kinase (ERK1/2) and p38, to define the mechanism of PAF-R-dependent effect on gemcitabine-mediated MVP release. To that end, PANC-1 cells were pretreated with inhibitors of either ERK1/2 (PD98059; 10 µM) or P38 (SB202190; 10 µM), followed by treatment with or without gemcitabine (0.1 mM) with negative and positive controls. Our studies demonstrate that inhibitors of both ERK1/2 and P38 pathways blocked gemcitabine-mediated MVP release (Figure 6), indicating the roles of ERK1/2 and p38 in this event. The schematic representation of the working model is shown in Figure 7.

## 3. Discussion

As pancreatic cancer-associated mortality is on rise, several novel targets/approaches are being explored to enhance the treatment effectiveness for this malignancy. Considering the important roles of PAF-R in augmenting the growth and/or impeding the efficacy of cancer therapies in several experimental tumor model systems [3,4,5,6,7,8,9,10,11], the current studies determined the potential role and mechanism of functional PAF-R in mediating gemcitabine-induced MVP release in pancreatic cancer cells. MVPs are heterogeneous, membrane-bound bioactive extracellular vesicles which are released from the surface of various cell types, including tumor cells in response to stimuli, including anti-tumor agents [14,15,16,17]. Based upon the origins/cell types, MVPs have been referred to as shedding vesicles, ectosomes, oncosomes, shedding bodies, and microparticles. These MVPs have been implicated in mediating the biological activities of cells due to their ability to carry bioactive components, including lipids [12,13,14,15,16,17]. 

Our first studies using PAF-R-expressing PANC-1 and -deficient Hs766T pancreatic cancer cells demonstrated that exposure of gemcitabine, similar to a known PAF-R agonist, CPAF, induces the release of MVPs in a PAF-R-dependent manner. Nevertheless, treatment with PMA induced MVP release from both PANC-1 and Hs766T cells, confirming the importance of PAF-R in gemcitabine-induced MVP secretion. No further increase in MVPs was noted with higher doses of gemcitabine. Although, the exact reason for this discrepancy is not clear, it is possible that the higher doses of gemcitabine, due to increased PAF-agonist production, could desensitize the PAF-R, similar to that observed in another report of CPAF treatment in PAF-R-expressing HaCaT cells [17]. These results are consistent with the recent reports from our group, that exposure to pro-oxidative stressor, such as UVB radiation, resulted in significant levels of MVP release from epidermal HaCaT and KBP cells in a PAF-R-dependent manner [16,17]. Similar to our current studies, this UVB-mediated effect on MVP release was mimicked by CPAF [16,17]. These findings also indicate that agents with pro-oxidative stressor properties can induce MVP release from various cells, yet these effects could be mediated via a PAF-R-dependent manner. 

To further confirm the role of the PAF-R in gemcitabine-mediated MVP release, our studies demonstrate that PAF-R blockade via a known PAF-R antagonist, WEB2086, significantly attenuated MVP release by gemcitabine, similar to what is observed with CPAF treatment. However, WEB2086 did not attenuate PMA-induced MVP release, indicating the involvement of the PAF-R in gemcitabine-mediated effects. Multiple studies support our findings that blockade of the PAF-R via specific antagonists, including WEB2086, attenuate PAF-R-dependent effects of various stimuli, including therapeutic agents, in several model systems [5,7,10,29,30,33]. 

The biogenesis underlying MVP formation and secretion are governed by multiple pathways, including the one dependent on lipid raft composition, and the activity of aSMase [34,35]. The aSMase hydrolyzes sphingomyelin to the sphingolipid ceramide which, upon activation of the MAPK (i.e., p38) pathway, induces the translocation of aSMase to the plasma membrane, resulting in the shedding/release of MVPs [34,35]. Imipramine, a member of tricyclic antidepressants (TCA), which belongs to the dibenzoazepine group, has been shown to inhibit aSMase via inducing its degradation and, thus, inhibition of MVP generation [36,37]. To that end, our next studies determine the effects of imipramine in gemcitabine-mediated MVP release, and demonstrate that pretreatment of imipramine blocked not only gemcitabine, but also CPAF- and PMA-mediated MVP release from pancreatic cancer cells, indicating the role of aSMase in this effect. 

Given that pro-oxidative stressors, including UVB and therapeutic agents, generate PAF-R agonists, which can be quantitatively accessed via measuring IL-8 secretion as a surrogate marker [1,2,3,4,5,6,7], and that these PAF-R agonists are transported via MVPs [16,17], in the current study, we wondered if gemcitabine-released MVPs contain these metabolically labile PAF-R agonists. Using this well-established approach, our studies demonstrate that the lipid extracts from gemcitabine-treated MVPs from PANC-1 cells contain measurable amounts of PAF-R agonists, comparable to CPAF directly added to KBP cells, compared to MVPs from vehicle-treated cells. 

Given the intriguing roles of MAPK pathways, in particular, p38 MAPK activation in aSMase-mediated MVP release [35], and ERK1/2 pathway in mediating PAF-R-dependent effects in response to various stimuli [33,38,39,40], we sought to define the mechanism of PAF-R-dependent gemcitabine-induced MVP release, by evaluating the roles of p38 and ERK1/2 pathways. Our studies demonstrate that pretreatment of PANC-1 cells with inhibitors of p38 and ERK1/2 significantly attenuated gemcitabine-induced release of MVP. These findings indicate the involvements of both p38 and ERK1/2 pathways as potential mechanisms of PAF-R-gemcitabine-mediated MVP release. 

These studies are consistent with the recent report demonstrating that gemcitabine treatment of pancreatic cancer cells triggers MVP release in various proportions/amounts, which correlated with the abilities of various cells to resist gemcitabine effects at different sensitivities [41]. Similar to our studies that only PANC-1 cells, but not Hs766T cells, were able to release MVPs in response to gemcitabine, it is possible that some of the pancreatic cancer cells which exhibited increased sensitivity to gemcitabine-mediated MVP release in this study [41], were PAF-R-expressing, and others showing less sensitivity to gemcitabine effects were PAF-R-deficient. 

In summary, the current studies highlighted the potential role, and mechanism, of the PAF-R in gemcitabine-mediated MVP release in pancreatic cancer cells. Importantly, given the crucial roles of PAF-R in modulating the efficacy of therapeutic agents, these studies provide the rationale of evaluating chemotherapeutic MVP-based approaches to combat pancreatic cancer. 

## 4. Materials and Methods

### 4.1. Reagents

The RNA extraction kit was purchased from Qiagen Sciences (Germantown, MD, USA). The SuperScript™ FirstStrand cDNA Synthesis kit, and SYBR green PCR reagent were purchased from Invitrogen Life Technologies (Carlsbad, CA, USA). The PAF-R and GAPDH primers were purchased from SABiosciences (Valencia, CA, USA). The PAF-R antagonist, WEB2086, CPAF, and imipramine were purchased from Cayman Chemicals Co. (Ann Arbor, MI, USA). The human IL-8 ELISA kit was from R&D Systems (Minneapolis, MN). All other reagents were purchased from Sigma-Aldrich (St. Louis, MO, USA).

### 4.2. Cell Culture

Human PANC-1 and Hs766T pancreatic cancer cells were procured from ATCC (Manassas, VA, USA) and cultured in DMEM media (Life Technologies, Grand Island, NY, USA) supplemented with 10% fetal bovine serum (Corning, NY, USA), and a 100 μg/mL mixture of penicillin and streptomycin (Lonza, Walkersville, MD, USA). Similarly, KBP and KBM cells were maintained in DMEM media supplemented with 10% fetal bovine serum and 100 μg/mL mixture of penicillin and streptomycin, as previously described by us [42]. 

### 4.3. Reverse Transcription-Quantitative PCR (RT-qPCR)

The mRNA expression of PAF-R was analyzed in the human PANC-1 and Hs766T cells using RT-qPCR, and the expression levels were normalized with GAPDH, as described previously [3,30]. The KBP and KBM cells were used as positive and negative controls. Briefly, the cells were homogenized using an RLT buffer containing β-mercaptoethanol (Sigma-Aldrich, St. Louis, MO, USA). The total RNA was extracted using a Qiagen RNeasy kit according to the manufacturer’s instructions. The purified RNA was quantified using a NanoDrop 2000 (Thermo Fisher Scientific, Inc., Lafayette, CO, USA) and reverse transcribed with a SuperScript cDNA Synthesis kit containing random hexamers. The cDNA was analyzed for the PAF-R mRNA using a SYBR green-based, quantitative fluorescent PCR method. The fluorescence was detected using a StepOne Real-Time PCR machine (Applied Biosystems, Foster City, CA, USA). The quantification of each PCR product was normalized to GAPDH using the 2^−^*^ΔΔ^*^*C*t^ method.

### 4.4. Assessment of Microvesicle Particle Release

MVPs were collected from culture medium as previously described [16,17]. In brief, the PANC-1 and Hs766T cells were treated with or without gemcitabine (0.1, 0.5, and 1 mM) and incubated for 4 h. These cells treated with CPAF (100 nM) or PMA (100 nM) served as positive controls. After 4 h, the culture medium was collected, and centrifuged at 2000× *g* for 20 min to remove cells and debris. The supernatant was collected, and subjected to centrifugation at 20,000× *g* for 70 min, and the resulting pellet was the isolated MVP. The concentration of the MVPs was detected by using a NanoSight NS300 instrument (NanoSight Ltd.), and regarded as the number of MVPs per mL of culture medium, as described [16,17]. In separate experiments, the cells were pretreated either with PAF-R antagonist (WEB2086; 10 µM) or inhibitors of MVP release (Imipramine; 20 µM) or ERK1/2 (PD98059; 10 µM), or P38 (SB202190; 10 µM) pathway, followed by isolation and measurement of MVPs as described. 

### 4.5. Measurement of Interleukin 8 (IL-8) Release

PANC-1 cells were treated with or without PMA (100 nM) or gemcitabine (0.1 mM), and cultured for 4 h followed by lipid extraction. KBP and KBM cells were treated with these lipid extracts, and with CPAF (1 nM), and incubated for 6 h. Supernatants were collected, and IL-8 release in the supernatant (as a surrogate marker of PAF-agonists) was measured by human IL-8 ELISA kit (R & D Systems), as previously described [2,4]. 

### 4.6. Statistical Analysis

Statistical analysis was assessed by GraphPad Prism software version 5.0 (GraphPad software, San Diego, CA, USA). All in vitro experiments were repeated, independently, at least three times. Data were analyzed by Student’s *t*-test or one-way ANOVA with post hoc Bonferroni’s multiple comparison tests. *p* < 0.05 was considered to indicate a statistically significant difference.

## Abbreviations

PAF	platelet-activating factor
PAF-R	platelet-activating factor-receptor
MVP	microvesicle particles
CPAF	1-hexadecyl-2-*N*-methylcarbamoyl glycerophosphocholine
GEM	gemcitabine
PMA	phorbol 12-myristate 13-acetate
aSMase	acid sphingomyelinase
MAPK	mitogen-activated protein kinase
ERK1/2	extracellular signal-regulated kinase
